# Past-month alcohol use in relation to the COVID-19 lockdown in 13 Indian states and union territories: gender, socioeconomic status, and state alcohol policy differences

**DOI:** 10.3389/fpubh.2026.1915064

**Published:** 2026-09-07

**Authors:** Md Zubab Ibne Moid, Abhishek Ghosh, Shinjini Choudhury, Jeremy Bray

**Affiliations:** 1 Center for Youth, Family and Community Partnerships, University of North Carolina at Greensboro, Greensboro, NC, United States; 2 Department of Psychiatry, Postgraduate Institute of Medical Education and Research, Chandigarh, India; 3 Institute of Neurosciences, Kolkata, West Bengal, India; 4 Department of Economics, University of North Carolina at Greensboro, Greensboro, NC, United States

**Keywords:** alcohol use, Bayesian logistic regression, COVID-19, gender, India

## Abstract

**Objectives:**

This study examined whether past-month alcohol use after the lockdown was higher or lower than expected based on the pattern observed before the lockdown in 13 Indian states and Union Territories, with particular attention to differences by gender, socioeconomic status, and state alcohol-policy context.

**Methods:**

We analysed National Family Health Survey data from 13 Indian states and union territories surveyed before and after the lockdown period. The primary outcome was any past-month alcohol use vs. no alcohol use. To address sparse alcohol-use categories, especially among women, we used Bayesian aggregated binomial logistic regression models estimated separately for women and men. Models included time trend, post-lockdown period, subgroup interactions, and state-level random intercepts. Subgroup-specific posterior odds ratios, predicted probabilities, 95% credible intervals, and posterior probabilities were estimated for age, residence, education, wealth, marital status, religious alcohol norms, scheduled tribe status, and state alcohol-policy category.

**Results:**

Among women, the odds of past-month alcohol use after the lockdown were higher than expected based on the earlier pattern. The posterior odds ratio for women was 1.83, with a 95% credible interval of 1.42–2.20, and the posterior probability of a positive association was approximately 1.00. Among men, the estimated association was smaller but still had high posterior probability, with a posterior odds ratio of 1.09, a 95% credible interval of 1.01–1.16, and a posterior probability of positive association of 0.99. Subgroup results suggested that positive associations among women were more evident among older women and socioeconomically disadvantaged groups, including rural women and women with lower education or wealth. Among men, subgroup patterns were smaller and more heterogeneous.

**Conclusion:**

The association between the post-lockdown period and past-month alcohol use differed between women and men in the 13 included states and union territories. The strongest positive association was observed among women, particularly women in socioeconomically disadvantaged subgroups such as rural women and women with lower education or wealth. These findings represent adjusted associations and should not be interpreted as causal effects of the national lockdown.

## Introduction

Alcohol use is a major public health concern in India, where approximately 160 million people reported past-year alcohol use, and more than one-third of users were estimated to have harmful use (1). Although alcohol use is substantially more common among men than women in India, gender differences in alcohol use are shaped by social norms, stigma, access, and differences in alcohol-related health risks. Women may experience alcohol-related harm at lower levels of consumption and may progress more rapidly to adverse physical consequences than men (2). These gendered vulnerabilities may also intersect with socioeconomic disadvantage. The alcohol-harm paradox suggests that socioeconomically disadvantaged groups can experience higher levels of alcohol-related illness and mortality even when they consume similar or lower amounts of alcohol than more advantaged groups (3). These differences are especially important in low- and middle-income countries, where alcohol-attributable harms are disproportionately high and public health resources for prevention and treatment are often limited (4, 5). Therefore, examining alcohol use through both gender and socioeconomic lenses is important for understanding alcohol-related vulnerability in India.

The COVID-19 pandemic and the national lockdown disrupted alcohol availability, mobility, income, social interaction, and access to health services in India. Although alcohol sales were formally restricted during the lockdown, state-level responses varied, and access to alcohol did not disappear completely because of differences in enforcement, informal supply, illicit production, and smuggling (6, 7). India's federal structure gives states and union territories substantial authority over alcohol regulation, including sale restrictions, taxation, legal drinking age, and prohibition policies, creating important regional variation in alcohol availability and control (8).

Previous literature suggests that such disruptions can influence drinking beyond the strict lockdown period through several pathways. Sudden restrictions on alcohol availability may lead to withdrawal, craving, black-market purchasing, or substitution through illicit and non-consumable alcohol, while reopening may generate rebound demand after a period of constrained access (6, 9–11). Pandemic-related stress, income loss, social isolation, and disruption of health and addiction services may also increase drinking as a coping response, particularly among socioeconomically vulnerable groups (10, 11). At the same time, evidence from the pandemic literature shows heterogeneous changes in alcohol use, with some groups reducing consumption because of lower availability, fewer social occasions, or economic constraints, while others increased drinking frequency, heavy drinking, or risky drinking, especially those experiencing stress or drinking to cope (12–14). Thus, lockdown alcohol-policy context may be relevant for post-lockdown drinking not only through direct restrictions on sales, but also through the broader disruption and reopening of alcohol markets, coping behavior, and informal access.

The National Family Health Survey offers a unique opportunity to examine alcohol use before and after the national lockdown period because NFHS-5 data collection was interrupted by the COVID-19 lockdown and resumed afterward. However, the survey alcohol item measures use in the past month, meaning that the post-lockdown responses should not be interpreted as alcohol use during the strict lockdown itself. Instead, the data allow comparison of past-month alcohol use between the pre-lockdown survey period and the post-lockdown survey period. NFHS-5 used a stratified, multistage sampling design and collected alcohol-use information from women aged 15–49 years and men aged 15–54 years, allowing gender-stratified analysis of changes in alcohol use across demographic, socioeconomic, and state-policy subgroups (15).

This study examined whether past-month alcohol use during the post-lockdown period differed from the level expected based on the earlier pattern of alcohol use, and whether this association differed by gender, socioeconomic status, and state alcohol-policy context. We hypothesized that post-lockdown associations with alcohol use would differ between women and men because of differences in baseline alcohol-use prevalence, social acceptability, access patterns, and alcohol-related risks. We also hypothesized that associations would vary across residence, education, wealth, marital status, religious alcohol norms, scheduled tribe status, and state alcohol-policy categories. To improve interpretability and address sparse drinking-frequency categories, especially among women, we focused on any past-month alcohol use vs. no alcohol use and estimated gender-stratified Bayesian binomial models.

## Methods

### Data source and analytic sample

We used data from the National Family Health Survey (NFHS), combining NFHS-4 and NFHS-5 observations from the 13 states and Union Territories that had NFHS-5 survey observations both before and after the national COVID-19 lockdown. NFHS-4 provided a pre-pandemic reference period, while NFHS-5 included observations collected before and after the national COVID-19 lockdown period. Survey period was classified as NFHS-4, NFHS-5 pre-lockdown, and NFHS-5 post-lockdown. NFHS-4 was conducted in 2015–2016. NFHS-5 was conducted in 2019–2021. India's national COVID-19 lockdown began on March 25, 2020 and continued in phases through May 31, 2020, with reopening beginning afterward. NFHS-5 interviews conducted on or before March 24, 2020, were classified as pre-lockdown observations, while interviews conducted after June 1, 2020, were classified as post-lockdown observations. Interviews conducted during the intervening lockdown period were excluded.

The analysis was restricted to 13 states and Union Territories with NFHS-5 observations in both the pre-lockdown and post-lockdown survey periods: Arunachal Pradesh, Chhattisgarh, Delhi, Haryana, Jharkhand, Madhya Pradesh, Odisha, Puducherry, Punjab, Rajasthan, Tamil Nadu, Uttar Pradesh, and Uttarakhand. States and Union Territories were included if NFHS-5 fieldwork contained observations both before and after the national lockdown interruption. This criterion was determined by the timing and resumption of NFHS-5 fieldwork rather than by the observed prevalence of alcohol use. Nevertheless, the included states may differ from excluded states in geography, baseline alcohol use, pandemic conditions, fieldwork timing, and policy context. Therefore, the findings should be interpreted as applying to these included states and Union Territories rather than as nationally representative estimates for all of India.

The primary analysis included observations from NFHS-4, NFHS-5 pre-lockdown, and NFHS-5 post-lockdown. The analysis distinguished the underlying time trend from the additional association specific to the post-lockdown period. State-level random intercepts accounted for baseline differences across states and Union Territories. The analytic sample included women aged 15–49 years and men aged 15–54 years, consistent with the NFHS sampling design.

### Outcome variable

The primary outcome was any alcohol consumption during the month preceding the interview. The National Family Health Survey categorized alcohol-use frequency as almost every day, about once a week, less than once a week, or no alcohol use. The first three categories were combined into a binary measure of any past-month alcohol use vs. no past-month alcohol use. This binary measure improved interpretability and reduced instability associated with sparse drinking-frequency categories, particularly among women and smaller demographic subgroups.

The same outcome definition was applied across NFHS-4, NFHS-5 pre-lockdown, and NFHS-5 post-lockdown. Because the survey question referred to the month preceding each respondent's interview, observations from the NFHS-5 post-lockdown period should not be interpreted as measuring alcohol consumption during the strict national lockdown.

### Explanatory variables

We included two variables representing survey timing, with each serving a different purpose. First, a time-trend variable took successive values of 0 for NFHS-4, 1 for NFHS-5 pre-lockdown, and 2 for NFHS-5 post-lockdown. This variable represented the underlying direction of change in alcohol use across the three survey periods. Second, a post-lockdown indicator took the value 1 for NFHS-5 post-lockdown observations and 0 for observations from the two earlier periods. After accounting for the time trend, this indicator estimated whether alcohol use during the NFHS-5 post-lockdown period was higher or lower than expected if the change observed between NFHS-4 and NFHS-5 pre-lockdown had continued. Thus, the time trend represented the underlying pattern across survey periods, while the post-lockdown indicator represented the additional departure from that pattern during the NFHS-5 post-lockdown period.

Covariates included age group, types of residence (rural vs. urban), education (no education vs. primary vs. secondary vs. higher), economic status (poorest to richest quintiles), marital status (never in union vs. married vs. separated/divorced/widowed), scheduled tribe (yes vs. no), and religion (prohibit vs. do not prohibit alcohol use). Urban/rural classification was based on census data. Education was categorized by years of schooling: none, 1–5 years, 6–12 years, and more than 12 years. Economic quintiles used a composite wealth index based on household assets, housing, and access to services.

Thirteen states and Union Territories were grouped into three ordered categories according to the primary dimension of the degree of restriction on the legal, physical availability of alcohol during and immediately after the national lockdown: temporary prohibition (no legal sales during the initial lockdown phases), restricted sales (sales permitted but constrained, e.g., limited hours or days, e-token systems, or online and home delivery), and relaxed policies (legal availability broadly maintained with minimal restrictions). We selected legal availability as the organizing dimension because the study outcome is any past-month alcohol use, for which access to a legal supply is the most proximate policy-relevant determinant. Although most states also modified excise duties or introduced COVID-specific cesses during this period (Table 1), these fiscal measures were near-universal across categories and therefore were not used to define the classification.

**Table 1:** Classification of Indian states based on changes in alcohol policies during the pandemic.

Policy classification	State/union territory	Policy description	Excise duty changes
States with Temporary Prohibition	Tamil Nadu	Initial prohibition, later relaxed.	Increased prices of alcohol sold through TASMAC stores.
	Andhra Pradesh	Temporary prohibition during initial lockdown phases.	Increased excise duty significantly and imposed an additional COVID tax.
	Kerala	Temporary prohibition, later relaxed with strict regulations.	Increased excise duty by about 10%.
States with Restricted Sales	Maharashtra	Limited hours and days for sale, allowed online sales.	Raised excise duty moderately on various categories of alcoholic beverages.
	Delhi	E-tokens for alcohol purchase to maintain social distancing.	Implemented a 70% ‘special corona fee' on alcohol in May 2020.
	Karnataka	Restricted timings and days, followed by a gradual relaxation.	Raised excise duty on all types of liquor.
	West Bengal	E-tokens for alcohol purchase and home delivery options.	Imposed a 30% tax hike on liquor sales.
	Uttar Pradesh	Limited timings and days, followed by relaxation.	Increased excise duty on alcohol significantly.
	Haryana	Limited timings, days, and introduction of home delivery.	Introduced a ‘COVID cess' on alcohol.
	Punjab	Limited timings and days with strict regulations.	Increased excise duty by 10–15%.
	Chhattisgarh	Online sales and home delivery options.	Introduced a 10% additional excise duty as a COVID-19 surcharge.
	Assam	E-tokens and home delivery initiatives.	Imposed a moderate increase in excise duty on liquor sales.
	Arunachal Pradesh	The state relaxed rules to facilitate the sale and distribution of alcohol, allowing for easier access.	Imposed a 25% cess on all alcoholic beverages, including Indian Made Foreign Liquor (IMFL), beer, imported alcoholic beverages, and wine, starting from May 2020.
States with Relaxed Policies (Post Initial Lockdown)	Jharkhand	Kept liquor stores open as essential services. The state implemented measures to ensure the availability of alcohol while maintaining COVID-19 safety protocols.	Jharkhand did not implement any significant new taxes specifically in response to COVID-19.
	Puducherry	Classified liquor stores as essential services, allowing them to operate during the lockdown.	Did not introduce significant new alcohol taxes.
	Uttarakhand	Deemed liquor stores as essential services, allowing them to remain open during lockdowns.	Did not significantly alter its alcohol tax rates during the pandemic.
	Rajasthan	Gradual relaxation with specific timings.	Implemented a 10–20% increase in excise duty on alcohol.
	Odisha	Relaxed sales with specific timings.	Imposed an additional COVID tax on alcohol sales.
	Telangana	Initially strict, later relaxed with timings.	Increased excise duty, but to a moderate extent.
	Madhya Pradesh	Gradual relaxation post initial lockdown.	Imposed a moderate increase in excise duty.
	Goa	Limited timings, later relaxed.	Maintained relatively low excise duties with minor adjustments.
	Himachal Pradesh	Limited hours initially, later relaxed.	Implemented a moderate increase in excise duty.
	Chandigarh	Managed alcohol sales with safety measures.	Implemented a moderate increase in excise duty.
	Daman and Diu	Managed alcohol sales with safety measures.	Maintained low excise duties with minor adjustments.

### Statistical analysis

Because past-month alcohol use was uncommon in several subgroups, particularly among women, we used Bayesian binomial logistic regression rather than relying only on conventional maximum-likelihood logistic regression. Sparse binary outcomes and small numbers of events within cross-classified covariate cells can make frequentist logistic regression unstable, especially when subgroup interactions are included. In such settings, maximum-likelihood estimates may be biased, have very wide or unreliable standard errors, or fail because of complete or quasi-complete separation (16, 17). A Bayesian approach helps address these issues by using prior distributions to regularize model estimates, stabilize estimation in sparse cells, and produce direct probability statements through posterior credible intervals and posterior probabilities (18). This approach was therefore appropriate for estimating gender-stratified and subgroup-specific associations while reflecting uncertainty from sparse alcohol-use counts.

We aggregated respondents into covariate cells and modeled the number reporting any past-month alcohol use out of the total number in each cell using Bayesian binomial logistic regression models estimated separately for women and men. Covariate cells were defined by survey period, state or Union Territory, age group, residence, education, household wealth, marital status, religious alcohol norms, scheduled tribe status, and state alcohol-policy category. Models included the time trend across NFHS-4, NFHS-5 pre-lockdown, and NFHS-5 post-lockdown; the NFHS-5 post-lockdown indicator; subgroup covariates; interactions between the post-lockdown indicator and subgroup characteristics; and state-level random intercepts. All three survey periods therefore contributed to estimation of the posterior distributions.

All population-level regression coefficients were assigned normal prior distributions with a mean of 0 and a standard deviation of 1.5. The model intercept was assigned a Student's t prior distribution with 3 degrees of freedom, a location of 0, and a scale of 2.5. The standard deviation of the state-level random intercept was assigned an exponential prior distribution with a rate of 1. These weakly informative priors were centered on no association while allowing the observed data to support positive or negative associations. Posterior distributions were estimated using two Markov chain Monte Carlo chains with 2,000 iterations per chain, including 1,000 warm-up iterations. Posterior means were used as point estimates, and 95% credible intervals were calculated from the 2.5th and 97.5th percentiles of the retained posterior draws.

Subgroup-specific associations were estimated using marginal standardization over the NFHS-5 post-lockdown analytic population. For each posterior draw, predicted probabilities were calculated with the post-lockdown indicator set first to 0 and then to 1, while the time trend was held at its NFHS-5 post-lockdown value and the remaining characteristics were retained at their observed values. Predictions were weighted by the number of respondents represented by each covariate cell. We report posterior means, 95% credible intervals, probability differences, probability ratios, odds ratios, and posterior probabilities. Probability differences describe the absolute difference between the two predicted probabilities, while probability ratios describe their relative difference. Odds ratios compare the corresponding odds of any past-month alcohol use. The posterior probability of a positive association was calculated as the proportion of retained posterior draws in which the predicted probability with the post-lockdown indicator set to 1 exceeded the corresponding probability with the indicator set to 0. The complete Bayesian model specification and statistical equations are presented in Supplementary Appendix 1. Analyses were conducted in *R* using the brms package, and estimates were interpreted as associations rather than causal effects. Complete posterior results are reported in Supplementary Appendix 2. The Bayesian models used unweighted respondent counts and did not incorporate the NFHS sampling weights, stratification, or clustering. The estimates should therefore be interpreted as model-based associations within the analytic sample rather than as design-weighted population estimates.

## Results

### Descriptive statistics

The analytic sample across NFHS-4, NFHS-5 pre-lockdown, and NFHS-5 post-lockdown periods included 461,552 respondents from NFHS-4, 179,316 from the NFHS-5 pre-lockdown period, and 258,812 from the NFHS-5 post-lockdown period. Across survey periods, most respondents were women (86.9% in NFHS-4, 88.0% in NFHS-5 pre-lockdown, and 87.7% in NFHS-5 post-lockdown), rural residents (69.2%, 74.5%, and 74.8%, respectively), and married or living with a partner (70.3%, 69.5%, and 68.7%, respectively). The mean age was similar across periods: 29.82 years (SD = 9.99) in NFHS-4, 30.18 years (SD = 10.08) in NFHS-5 pre-lockdown, and 30.34 years (SD = 10.08) in NFHS-5 post-lockdown. The distribution of education, wealth, and state alcohol-policy categories varied across survey periods. The share of respondents with no education declined from 28.7% in NFHS-4 to 24.1% in NFHS-5 pre-lockdown and 22.7% in NFHS-5 post-lockdown, while the share with secondary or higher education increased from 58.3 to 63.4 and 65.8%, respectively. Wealth composition also varied, with the poorest quintile representing 21.2%, 18.8%, and 23.5% of respondents across the three periods, respectively, and the richest quintile representing 21.5%, 20.3%, and 20.1%. State alcohol-policy categories showed larger differences: respondents from relaxed-policy states accounted for 46.9% of NFHS-4, 47.5% of NFHS-5 pre-lockdown, and 38.8% of NFHS-5 post-lockdown observations. The corresponding proportions from restricted-sales states were 45.7%, 47.6%, and 53.4%.

Overall past-month alcohol use was 6.3% in NFHS-4, 4.6% in the NFHS-5 pre-lockdown period, and 5.1% in the NFHS-5 post-lockdown period. Alcohol use was substantially higher among men than women in all periods. Among women, past-month alcohol use was 2.3% in NFHS-4, 1.6% in NFHS-5 pre-lockdown, and 2.0% in NFHS-5 post-lockdown. Among men, the corresponding percentages were 32.6%, 26.3%, and 26.7%, respectively. Values in Table 2 are presented as *n* (%) unless otherwise indicated; age is presented as mean (SD). (see Table 2)

**Table 2:** Descriptive characteristics of the analytic sample across NFHS-4, NFHS-5 pre-lockdown, and NFHS-5 post-lockdown periods.

Characteristic	Level	NFHS-4	NFHS-5 pre-lockdown	NFHS-5 post-lockdown
Total respondents		461,552	179,316	258,812
Age, mean (SD)		29.82 (9.99)	30.18 (10.08)	30.34 (10.08)
Sex	Male	60,556 (13.1)	21,578 (12.0)	31,889 (12.3)
Sex	Female	400,996 (86.9)	157,738 (88.0)	226,923 (87.7)
Age group	15-24	167,930 (36.4)	62,693 (35.0)	88,830 (34.3)
Age group	25-34	135,906 (29.4)	52,789 (29.4)	76,623 (29.6)
Age group	35-44	106,071 (23.0)	42,323 (23.6)	62,018 (24.0)
Age group	Women: 45–49 years; men: 45–54 years	51,645 (11.2)	21,511 (12.0)	31,341 (12.1)
Residence	Rural	319,214 (69.2)	133,578 (74.5)	193,682 (74.8)
Residence	Urban	142,338 (30.8)	45,738 (25.5)	65,130 (25.2)
Education	No education	132,574 (28.7)	43,128 (24.1)	58,680 (22.7)
Education	Primary	59,755 (12.9)	22,493 (12.5)	30,051 (11.6)
Education	Secondary	211,369 (45.8)	87,072 (48.6)	129,548 (50.1)
Education	Higher	57,854 (12.5)	26,623 (14.8)	40,533 (15.7)
Wealth quintile	Poorest	97,935 (21.2)	33,760 (18.8)	60,763 (23.5)
Wealth quintile	Poorer	92,674 (20.1)	39,522 (22.0)	52,890 (20.4)
Wealth quintile	Middle	87,164 (18.9)	36,242 (20.2)	47,387 (18.3)
Wealth quintile	Richer	84,764 (18.4)	33,335 (18.6)	45,776 (17.7)
Wealth quintile	Richest	99,015 (21.5)	36,457 (20.3)	51,996 (20.1)
Marital status	Never in union	121,754 (26.4)	48,572 (27.1)	71,930 (27.8)
Marital status	Married/Living together	324,454 (70.3)	124,707 (69.5)	177,837 (68.7)
Marital status	Divorced/Separated/Widowed	15,344 (3.3)	6,037 (3.4)	9,045 (3.5)
Religious alcohol norms	Permits	415,823 (90.1)	166,903 (93.1)	237,201 (91.6)
Religious alcohol norms	Prohibits	45,729 (9.9)	12,413 (6.9)	21,611 (8.4)
Scheduled tribe status	No tribe	406,984 (88.2)	163,208 (91.0)	221,956 (85.8)
Scheduled tribe status	Tribe	54,568 (11.8)	16,108 (9.0)	36,856 (14.2)
State alcohol-policy category	States with relaxed policy	216,388 (46.9)	85,245 (47.5)	100,347 (38.8)
State alcohol-policy category	States with temporary prohibition	34,034 (7.4)	8,691 (4.8)	20,331 (7.9)
State alcohol-policy category	States with restricted sales	211,130 (45.7)	85,380 (47.6)	138,134 (53.4)
Past-month alcohol use	No alcohol use	432,375 (93.7)	171,058 (95.4)	245,738 (94.9)
Past-month alcohol use	Any alcohol use	29,177 (6.3)	8,258 (4.6)	13,074 (5.1)
Past-month alcohol use among men	Any alcohol use	19,756 (32.6)	5,684 (26.3)	8,528 (26.7)
Past-month alcohol use among women	Any alcohol use	9,421 (2.3)	2,574 (1.6)	4,546 (2.0)

The oldest age group included women aged 45–49 years and men aged 45–54 years.

### Adjusted post-lockdown associations from Bayesian estimation

Table 3 presents adjusted odds ratios showing how the odds of any past-month alcohol use during the NFHS-5 post-lockdown period differed from the odds expected under continuation of the time trend, after accounting for respondent characteristics and state-level differences. Among women, the odds ratio of drinking was 1.83, with a 95% credible interval of 1.42–2.20, indicating higher odds of alcohol use in the post-lockdown period. The posterior probability of higher alcohol use was 1.00, meaning that essentially all posterior draws suggested an increase among women. Among men, the odds ratio was 1.09, with a 95% credible interval of 1.01–1.16, and the posterior probability of higher alcohol use was 0.99. The post-lockdown association was therefore smaller among men than among women.

**Table 3:** Adjusted post-lockdown odds ratio of past-month alcohol use relative to the expected time trend, by gender.

Gender	Adjusted odds ratio	95% credible interval
Women	1.83	1.42–2.20
Men	1.09	1.01–1.16

Table 4 presents subgroup-specific adjusted odds ratios showing how the odds of any past-month alcohol use during the NFHS-5 post-lockdown period differed from the odds expected under continuation of the time trend. Among women, post-lockdown odds of alcohol use were higher across most subgroups. The increase became larger with age, rising from an odds ratio of 1.11 among women aged 15–24 years (95% CrI: 0.85–1.38) to 2.23 among women in the oldest age group (95% CrI: 1.71–2.70). Rural women showed a larger increase than urban women (OR: 1.88, 95% CrI: 1.48–2.26 vs. OR: 1.50, 95% CrI: 0.96–1.92). By education, the largest increase was observed among women with no education (OR: 2.06, 95% CrI: 1.63–2.50), followed by women with primary education (OR: 1.72, 95% CrI: 1.31–2.14) and secondary education (OR: 1.58, 95% CrI: 1.18–1.94); the estimate for women with higher education was smaller and less precise (OR: 1.25, 95% CrI: 0.80–1.64). A similar socioeconomic pattern was observed by wealth: women in the poorest quintile had higher post-lockdown odds (OR: 1.93, 95% CrI: 1.53–2.34), while the estimate for women in the richest quintile was smaller and more uncertain (OR: 1.52, 95% CrI: 0.98–2.01). Post-lockdown odds were also higher among married women (OR: 1.93, 95% CrI: 1.49–2.33) and divorced, separated, or widowed women (OR: 2.24, 95% CrI: 1.67–2.80), but not clearly higher among women never in union (OR: 1.02, 95% CrI: 0.77–1.26). By state alcohol-policy category, the largest association was observed among women in restricted-sales states (OR: 2.38, 95% CrI: 2.13–2.65), followed by relaxed-policy states (OR: 1.45, 95% CrI: 1.29–1.62). Post-lockdown odds were lower among women in states with temporary prohibition (OR: 0.55, 95% CrI: 0.28–0.92).

**Table 4:** Subgroup-specific adjusted post-lockdown odds ratio of past-month alcohol use relative to the expected time trend.

Level	Women OR (95% CrI)	Men OR (95% CrI)
Age group		
15–24	1.11 (0.85–1.38)	0.90 (0.81–0.99)
25–34	1.88 (1.48–2.28)	1.04 (0.95–1.13)
35–44	1.93 (1.50–2.34)	1.18 (1.09–1.29)
Women: 45–49 years; men: 45–54 years	2.23 (1.71–2.70)	1.26 (1.15–1.37)
Residence		
Rural	1.88 (1.48–2.26)	1.12 (1.04–1.20)
Urban	1.50 (0.96–1.92)	0.98 (0.90–1.07)
Education		
No education	2.06 (1.63–2.50)	1.13 (1.02–1.24)
Primary	1.72 (1.31–2.14)	1.26 (1.14–1.39)
Secondary	1.58 (1.18–1.94)	1.09 (1.01–1.17)
Higher	1.25 (0.80–1.64)	0.91 (0.82–1.01)
Wealth quintile		
Poorest	1.93 (1.53–2.34)	1.24 (1.14–1.35)
Poorer	1.62 (1.25–1.98)	1.10 (1.00–1.20)
Middle	2.02 (1.39–2.50)	1.10 (1.00–1.20)
Richer	1.79 (1.12–2.31)	0.92 (0.84–1.01)
Richest	1.52 (0.98–2.01)	1.00 (0.90–1.10)
Marital status		
Never in union	1.02 (0.77–1.26)	0.94 (0.86–1.02)
Married/living together	1.93 (1.49–2.33)	1.14 (1.06–1.23)
Divorced/separated/ widowed	2.24 (1.67–2.80)	1.30 (1.04–1.60)
Religious alcohol norms		
Permits	1.83 (1.42–2.20)	1.09 (1.02–1.17)
Prohibits	1.51 (0.72–2.66)	0.88 (0.72–1.07)
Scheduled tribe status		
No tribe	2.46 (1.78–2.94)	1.06 (0.99–1.13)
Tribe	1.23 (1.02–1.44)	1.22 (1.11–1.34)
State alcohol policy		
Relaxed policy	1.45 (1.29–1.62)	1.09 (1.01–1.18)
Temporary prohibition	0.55 (0.28–0.92)	0.89 (0.79–1.01)
Restricted sales	2.38 (2.13–2.65)	1.11 (1.03–1.20)

The oldest age group included women aged 45–49 years and men aged 45–54 years.

Among men, subgroup patterns were more modest and heterogeneous. Higher post-lockdown odds were concentrated among older men, increasing from OR: 1.18 among men aged 35–44 years (95% CrI: 1.09–1.29) to OR: 1.26 among men aged 45–54 years (95% CrI: 1.15–1.37). The odds were lower among men aged 15–24 years (OR: 0.90, 95% CrI: 0.81–0.99), while the estimate for men aged 25–34 years was closer to the null (OR: 1.04, 95% CrI: 0.95–1.13). Rural men showed higher post-lockdown odds (OR: 1.12, 95% CrI: 1.04–1.20), whereas the estimate for urban men was near the null (OR: 0.98, 95% CrI: 0.90–1.07). By education, the clearest increase was among men with primary education (OR: 1.26, 95% CrI: 1.14–1.39), while estimates for no education, secondary education, and higher education were smaller or uncertain. By wealth, higher post-lockdown odds were observed among men in the poorest (OR: 1.24, 95% CrI: 1.14–1.35), poorer (OR: 1.10, 95% CrI: 1.00–1.20), and middle wealth quintiles (OR: 1.10, 95% CrI: 1.00–1.20), but not among richer or richest men. Higher odds were also observed among married men (OR: 1.14, 95% CrI: 1.06–1.23), divorced, separated, or widowed men (OR: 1.30, 95% CrI: 1.04–1.60), scheduled tribe men (OR: 1.22, 95% CrI: 1.11–1.34), men in relaxed-policy states (OR: 1.09, 95% CrI: 1.01–1.18), and men in restricted-sales states (OR: 1.11, 95% CrI: 1.03–1.20). In contrast, the estimate for men in temporary-prohibition states was below 1 but remained uncertain (OR: 0.89, 95% CrI: 0.79–1.01).

## Discussion

This study found gender differences in the adjusted post-lockdown association with past-month alcohol use in the 13 included states and Union Territories. After accounting for the time trend across the three survey periods, women had 83% higher odds of past-month alcohol use than expected, while the corresponding increase among men was smaller at 9%. Subgroup analyses suggested that the increase among women was more evident in several socially and economically disadvantaged groups, including rural women, women with no education, and women in lower wealth groups. Among men, subgroup patterns were more heterogeneous, with higher post-lockdown alcohol use observed among older men, rural men, scheduled tribe men, and men in states with restricted sales. State alcohol-policy context was also associated with post-lockdown alcohol use. Among women, the largest increase was observed in restricted-sales states, while lower post-lockdown odds were observed in states with temporary prohibition. Among men, positive post-lockdown associations were observed in both relaxed-policy and restricted-sales states. These policy-context findings should be interpreted cautiously as associations rather than causal effects. These findings add to international evidence that alcohol-use patterns changed unevenly during the COVID-19 pandemic. Studies from Europe and the United States have reported heterogeneous changes in alcohol use during the pandemic, with overall reductions in some general-population samples but increases or persistent high-risk drinking among heavier drinkers and specific age or college-status groups (13, 19, 20). One study (21) found that women were more likely than men to report changes in drinking after COVID-19 restrictions, and that increased drinking was associated with higher educational attainment. Our findings suggest that aggregate trends may conceal important gender and socioeconomic differences. Although alcohol use remains substantially less common among women than men in India, the clearer post-lockdown increase among women is important because women may experience alcohol-related harms at lower levels of consumption and may face greater stigma and barriers to treatment (2).

The subgroup findings suggest that post-lockdown changes in alcohol use may have intersected with socioeconomic vulnerability. Women with lower education, rural residence, and lower wealth showed stronger evidence of increased alcohol use. This pattern is important because lower socioeconomic status is associated with greater alcohol-attributable harm, even when consumption levels are similar or lower than in more advantaged groups (22, 23). Lockdown-related restrictions increased economic insecurity, social isolation, household stress, and caregiving demands, particularly for women, while prior research shows that emotional distress and drinking to cope are important pathways through which crises can increase alcohol use (24–27). The findings therefore suggest that public health responses should not focus only on overall alcohol prevalence, but should also monitor alcohol-use changes among socioeconomically vulnerable groups.

State alcohol-policy context was also associated with variation in post-lockdown alcohol use. However, these findings should be interpreted cautiously. State policy categories reflect formal rules and restrictions, not actual implementation, enforcement, informal supply, or illicit alcohol availability. Prior research from India showed that alcohol demand persisted despite formal restrictions and that access could continue through informal or illicit channels (6, 7). Therefore, the observed differences across policy categories should be interpreted as associations with policy context rather than evidence that specific lockdown alcohol policies caused changes in drinking. Because the analysis captures alcohol use after the strict lockdown period, these findings may reflect an emerging post-lockdown pattern rather than only an acute lockdown response.

The findings have implications for gender-responsive alcohol policy in the included states and Union Territories. Women's alcohol use is often less visible because of stigma, social norms, and lower baseline prevalence, but this should not lead to neglect in surveillance or treatment planning. Policies that restrict or liberalize alcohol access should consider gender-specific effects, particularly for women in disadvantaged socioeconomic groups. Strengthening screening, brief intervention, and referral pathways in primary care and community health settings may be especially important after major social disruptions.

This study has several limitations. First, the NFHS measures past-month alcohol use; therefore, the post-lockdown responses should not be interpreted as alcohol use during the strict national lockdown itself. Rather, the estimates describe alcohol use during the post-lockdown survey period relative to the level expected under continuation of the time trend across the three survey periods. The post-lockdown indicator captured interview timing and should not be interpreted as identifying the causal effect of the national lockdown. Differences between respondents interviewed before and after the lockdown may also reflect calendar time, seasonality, COVID-19 incidence, economic recovery, reopening conditions, state-specific restrictions, and changes in respondent composition. Second, the cross-sectional design prevents causal interpretation, and the results should be understood as associations between survey period and alcohol use. Third, the analysis included selected states and Union Territories and should not be interpreted as nationally representative of all India. Fourth, the state alcohol-policy classification is a state-level proxy that collapses several distinct dimensions: opening hours, prohibition, online and home delivery, excise and cess changes, enforcement, and reopening timings. These three were categories organized around the degree of restriction on legal alcohol availability. These dimensions did not always move together; for example, a state could restrict hours while expanding delivery access, or change excise taxation without altering physical availability. This aggregation may measurement error and likely misclassification of policy exposure, and does not capture enforcement, informal or illicit supply, or local implementation. The policy-context estimates should therefore be interpreted with particular caution. Fifth, the outcome was limited to any past-month alcohol use. NFHS does not provide detailed information on alcohol quantity, heavy episodic drinking, alcohol use disorder, treatment seeking, or alcohol-related harms. Finally, although the Bayesian analysis allowed estimation of subgroup-specific associations, residual confounding may remain.

In conclusion, the post-lockdown association with past-month alcohol use differed between women and men in the 13 included states and Union Territories. Women showed clearer evidence of higher post-lockdown alcohol use, especially in several socioeconomically disadvantaged subgroups, while changes among men were smaller and more heterogeneous. These findings support the need for gender-responsive alcohol surveillance and policy responses that consider socioeconomic vulnerability and the gap between formal alcohol policy and actual alcohol availability.

## Data Availability

The data underlying this article will be shared on reasonable request to the corresponding author, subject to institutional and ethical considerations.
