# Supplementary material for: Past-month alcohol use in relation to the COVID-19 lockdown in 13 Indian states and union territories: gender, socioeconomic status, and state alcohol policy differences
Source: Front Public Health. 2026 Sep 7;14:1915064. doi: 10.3389/fpubh.2026.1915064 (PMC13590371; doi:10.3389/fpubh.2026.1915064)
Supplement: Supplementary file 1 [file Table_1.docx]

Appendix 1: Bayesian Model Specification and Statistical Equations

## Model specification

Separate Bayesian binomial logistic regression models were estimated for women and men, with identical specifications. Respondents were aggregated into cells defined by survey period, state or Union Territory, age group, residence, education, household wealth, marital status, religious alcohol norms, scheduled tribe status, and state alcohol-policy category.

For cell $i$, $Y_{i}$ represents the number of respondents reporting any alcohol use during the month preceding the interview, $n_{i}$ represents the total number of respondents, and $p_{i}$ represents the probability of past-month alcohol use:

$$Y_{i}\mid n_{i},p_{i}\sim Binomial\left( n_{i},p_{i} \right).$$

The regression model was:

$$\mathrm{logit}\left( p_{i} \right)=ln\left( \frac{p_{i}}{1-p_{i}} \right)=\alpha+\beta_{T}T_{i}+\beta_{P}P_{i}+X_{i}^{'}\beta+P_{i}X_{i}^{'}\gamma+u_{s\left( i \right)}.$$

In this equation, $\alpha$ is the intercept, $T_{i}$ is the time trend, and $P_{i}$ is the NFHS-5 post-lockdown indicator. The vector $X_{i}$ contains age group, residence, education, household wealth, marital status, religious alcohol norms, scheduled tribe status, and state alcohol-policy category. The vector $\beta$ contains the coefficients for these characteristics, while $\gamma$ contains their interactions with the post-lockdown indicator. The term $u_{s\left( i \right)}$ is the random intercept for the state or Union Territory associated with cell $i$.

State-level random intercepts accounted for differences in baseline alcohol use across states and Union Territories and were assumed to follow:

$$u_{s}\mid\sigma_{u}\sim Normal\left( 0,\sigma_{u}^{2} \right),$$

where $\sigma_{u}$ represents between-state variation.

## Time trend and post-lockdown association

The time-trend and post-lockdown variables were assigned as follows:

NFHS-4: $T_{i}=0$ and $P_{i}=0$

NFHS-5 pre-lockdown: $T_{i}=1$ and $P_{i}=0$

NFHS-5 post-lockdown: $T_{i}=2$ and $P_{i}=1$

The time trend represented linear change across successive survey periods rather than annual calendar-time change. NFHS-4 and NFHS-5 pre-lockdown provided the two earlier periods used to estimate this trend.

For a given covariate profile, define:

$$C_{i}=\alpha+X_{i}^{'}\beta+u_{s\left( i \right)}.$$

The model-based log odds for NFHS-4 and NFHS-5 pre-lockdown were:

$$\eta_{i,NFHS4}=C_{i},$$

$$\eta_{i,pre}=C_{i}+\beta_{T}.$$

If the time trend continued into the third survey period without an additional post-lockdown departure, the expected log odds would be:

$$\eta_{i,expected}=C_{i}+2\beta_{T}.$$

After including the post-lockdown term and subgroup interactions, the estimated log odds were:

$$\eta_{i,post}=C_{i}+2\beta_{T}+\beta_{P}+X_{i}^{'}\gamma.$$

The subgroup-specific post-lockdown departure was therefore:

$$\delta_{i}=\eta_{i,post}-\eta_{i,expected}=\beta_{P}+X_{i}^{'}\gamma.$$

Thus, $\beta_{P}$ represents the post-lockdown association for the reference subgroup, while $\gamma$ represents differences in this association across subgroups. Covariate cells from all three survey periods jointly contributed to posterior estimation.

## Prior and posterior distributions

All population-level regression coefficients were assigned weakly informative normal prior distributions:

$$\beta_{T},\beta_{P},\beta,\gamma\sim Normal\left( 0,{1.5}^{2} \right).$$

The intercept was assigned:

$$\alpha\sim Student-t\left( 3,0,2.5 \right).$$

The standard deviation of the state-level random intercept was assigned:

$$\sigma_{u}\sim Exponential\left( 1 \right).$$

Let $\Theta$ represent all model parameters. The posterior distribution was obtained by combining the likelihood from the observed data with the prior distributions:

$$p\left( \Theta\mid Y \right)\propto\left[ \prod_{i} \mathrm{Binomial}\left( Y_{i}\mid n_{i},p_{i} \right) \right]p\left( \Theta\right).$$

Posterior distributions were estimated in Stan through the *brms* package in R. Two chains were run for 2,000 iterations each, including 1,000 warm-up iterations per chain, producing 2,000 retained posterior draws. The target acceptance probability was 0.95, and the maximum tree depth was 12.

## Standardized posterior estimates

Adjusted results were obtained using marginal standardization among respondents interviewed during the NFHS-5 post-lockdown period. The time trend was set to 2 under both comparison conditions. The post-lockdown indicator was set to 0 to estimate the probability expected under continuation of the time trend and to 1 to estimate the probability after including the post-lockdown association. Other respondent characteristics were retained at their observed values. State-level random intercepts were set to 0, and predictions were weighted by the number of respondents represented by each cell.

For subgroup $h$, posterior draw $m$, and post-lockdown setting $z$, the standardized predicted probability was:

$$\bar{p}_{h}^{\left( z,m \right)}=\frac{\sum_{i\in h} n_{i}p_{i}^{\left( z,m \right)}}{\sum_{i\in h} n_{i}}.$$

Here, $z=0$ represents the probability expected under continuation of the time trend, and $z=1$ represents the probability after including the post-lockdown association.

The probability difference was:

$$\mathrm{PD}_{h}^{\left( m \right)}=\bar{p}_{h}^{\left( 1,m \right)}-\bar{p}_{h}^{\left( 0,m \right)}.$$

The probability ratio was:

$$\mathrm{PR}_{h}^{\left( m \right)}=\frac{\bar{p}_{h}^{\left( 1,m \right)}}{\bar{p}_{h}^{\left( 0,m \right)}}.$$

The standardized odds ratio was:

$$\mathrm{OR}_{h}^{\left( m \right)}=\frac{\bar{p}_{h}^{\left( 1,m \right)}/\left[ 1-\bar{p}_{h}^{\left( 1,m \right)} \right]}{\bar{p}_{h}^{\left( 0,m \right)}/\left[ 1-\bar{p}_{h}^{\left( 0,m \right)} \right]}.$$

Posterior means were used as point estimates, and 95% credible intervals were defined by the 2.5th and 97.5th percentiles of the corresponding posterior distributions.

The posterior probability of a positive post-lockdown association was calculated as:

$$\Pr\left( \mathrm{PD}_{h}>0\mid Y \right)\approx\frac{1}{M}\sum_{m=1}^{M} I\left[ \mathrm{PD}_{h}^{\left( m \right)}>0 \right].$$

This represents the proportion of retained posterior draws in which the predicted probability after including the post-lockdown association was higher than the probability expected under continuation of the time trend.

The models used observed respondent counts and did not incorporate National Family Health Survey sampling weights or other elements of the complex survey design. Estimates were therefore interpreted as model-based associations within the analytic sample rather than as design-weighted population estimates.
