# Supplementary material for: Past-month alcohol use in relation to the COVID-19 lockdown in 13 Indian states and union territories: gender, socioeconomic status, and state alcohol policy differences
Source: Front Public Health. 2026 Sep 7;14:1915064. doi: 10.3389/fpubh.2026.1915064 (PMC13590371; doi:10.3389/fpubh.2026.1915064)
Supplement: Supplementary file 2 [file Table_2.docx]

**Appendix 2: Posterior Summaries and Model Diagnostics**

Posterior distributions were estimated separately for women and men using two Markov chain Monte Carlo chains with 2,000 iterations per chain. The first 1,000 iterations from each chain were used for warm-up, leaving 2,000 retained posterior draws for each model.

Tables A2.1 and A2.2 report the posterior mean, posterior standard deviation, 2.5th and 97.5th percentiles, R-hat, bulk effective sample size, and tail effective sample size for every model parameter. Regression coefficients and state-specific random intercepts are presented on the log-odds scale.

**Table A2.1. Posterior summaries for the Bayesian binomial logistic regression model among women**

| **Parameter** | **Posterior mean** | **Posterior SD** | **2.5th percentile** | **97.5th percentile** | **R-hat** | **Bulk ESS** | **Tail ESS** |
| --- | --- | --- | --- | --- | --- | --- | --- |
| Intercept | -4.2928 | 0.6709 | -5.4928 | -2.8864 | 1.0039 | 528.2 | 666.3 |
| Time trend | -0.4741 | 0.0263 | -0.5257 | -0.4221 | 1.0021 | 1908.2 | 1301.7 |
| Post-lockdown period | 0.3112 | 0.0969 | 0.1237 | 0.4992 | 0.9999 | 1168.6 | 1160.3 |
| Age 25-34 years (ref: 15-24) | 0.3762 | 0.0342 | 0.3110 | 0.4436 | 1.0024 | 1025.9 | 1351.5 |
| Age 35-44 years (ref: 15-24) | 0.6554 | 0.0365 | 0.5843 | 0.7249 | 1.0023 | 942.4 | 1172.0 |
| Age 45-54 years (ref: 15-24) | 0.7398 | 0.0418 | 0.6616 | 0.8221 | 1.0017 | 1071.9 | 1130.6 |
| Urban residence (ref: rural) | 0.0171 | 0.0350 | -0.0496 | 0.0883 | 0.9995 | 1790.9 | 1460.6 |
| Primary education (ref: no education) | -0.4805 | 0.0328 | -0.5469 | -0.4179 | 1.0001 | 1798.0 | 1608.6 |
| Secondary education (ref: no education) | -0.8557 | 0.0296 | -0.9122 | -0.7973 | 1.0015 | 1462.5 | 1454.8 |
| Higher education (ref: no education) | -0.2733 | 0.0517 | -0.3739 | -0.1746 | 0.9996 | 1621.8 | 1640.4 |
| Poorer wealth quintile (ref: poorest) | -0.7500 | 0.0283 | -0.8057 | -0.6948 | 1.0001 | 1724.9 | 1320.6 |
| Middle wealth quintile (ref: poorest) | -1.0448 | 0.0350 | -1.1112 | -0.9751 | 1.0004 | 1582.5 | 1562.9 |
| Richer wealth quintile (ref: poorest) | -1.1578 | 0.0446 | -1.2450 | -1.0711 | 1.0016 | 1459.6 | 1689.8 |
| Richest wealth quintile (ref: poorest) | -1.1217 | 0.0569 | -1.2342 | -1.0118 | 1.0005 | 1318.7 | 1588.8 |
| Married/living together (ref: never in union) | 0.1574 | 0.0372 | 0.0871 | 0.2312 | 1.0043 | 1053.0 | 1441.4 |
| Divorced/separated/widowed (ref: never in union) | 0.3629 | 0.0543 | 0.2550 | 0.4661 | 1.0021 | 1336.2 | 1326.5 |
| Religious norms prohibit alcohol (ref: permit) | -1.7800 | 0.1376 | -2.0546 | -1.5273 | 0.9995 | 2525.7 | 1708.3 |
| Scheduled tribe (ref: no tribe) | 1.3834 | 0.0253 | 1.3339 | 1.4323 | 1.0007 | 1992.1 | 1575.2 |
| Temporary-prohibition state (ref: relaxed policy) | -0.2248 | 1.1860 | -2.5601 | 2.0267 | 1.0006 | 1284.0 | 1187.1 |
| Restricted-sales state (ref: relaxed policy) | 0.0604 | 0.8419 | -1.6985 | 1.6784 | 1.0042 | 651.9 | 1013.6 |
| Post-lockdown x age 25-34 years | 0.3775 | 0.0729 | 0.2336 | 0.5176 | 1.0009 | 855.7 | 877.6 |
| Post-lockdown x age 35-44 years | 0.3453 | 0.0772 | 0.1937 | 0.4926 | 1.0003 | 889.8 | 1101.7 |
| Post-lockdown x age 45-54 years | 0.4747 | 0.0821 | 0.3162 | 0.6367 | 1.0002 | 876.3 | 1053.6 |
| Post-lockdown x urban residence | -0.1859 | 0.0697 | -0.3230 | -0.0499 | 1.0003 | 1647.4 | 1403.0 |
| Post-lockdown x primary education | -0.1408 | 0.0630 | -0.2639 | -0.0184 | 1.0005 | 1853.1 | 1557.9 |
| Post-lockdown x secondary education | -0.0821 | 0.0542 | -0.1863 | 0.0209 | 1.0022 | 1542.4 | 1450.7 |
| Post-lockdown x higher education | -0.2870 | 0.0995 | -0.4792 | -0.0899 | 1.0003 | 1804.6 | 1629.2 |
| Post-lockdown x poorer wealth quintile | -0.2959 | 0.0541 | -0.4013 | -0.1881 | 1.0001 | 1933.3 | 1393.3 |
| Post-lockdown x middle wealth quintile | -0.0740 | 0.0626 | -0.1992 | 0.0442 | 1.0043 | 1709.1 | 1458.4 |
| Post-lockdown x richer wealth quintile | -0.2218 | 0.0852 | -0.3912 | -0.0616 | 1.0009 | 1570.0 | 1555.5 |
| Post-lockdown x richest wealth quintile | -0.4570 | 0.1202 | -0.6985 | -0.2295 | 1.0004 | 1490.8 | 1493.8 |
| Post-lockdown x married/living together | 0.2910 | 0.0772 | 0.1419 | 0.4350 | 1.0011 | 974.7 | 1547.8 |
| Post-lockdown x divorced/separated/widowed | 0.4418 | 0.1035 | 0.2375 | 0.6400 | 1.0020 | 1178.2 | 1438.3 |
| Post-lockdown x religious norms prohibit alcohol | -0.5501 | 0.3122 | -1.2032 | 0.0434 | 1.0008 | 2203.9 | 1555.7 |
| Post-lockdown x scheduled tribe | -0.8128 | 0.0420 | -0.8931 | -0.7309 | 1.0004 | 1855.8 | 1428.2 |
| Post-lockdown x temporary-prohibition state | -1.1978 | 0.3015 | -1.8130 | -0.6359 | 1.0076 | 2213.9 | 1299.1 |
| Post-lockdown x restricted-sales state | 0.4740 | 0.0450 | 0.3893 | 0.5621 | 0.9994 | 2252.1 | 1665.7 |
| State-level random-intercept SD | 1.7738 | 0.3841 | 1.1894 | 2.7053 | 1.0096 | 604.3 | 977.8 |
| State random intercept: Arunachal Pradesh | 2.6839 | 0.6979 | 1.2572 | 4.0745 | 1.0008 | 637.2 | 897.0 |
| State random intercept: Chhattisgarh | 1.2536 | 0.6977 | -0.1511 | 2.6383 | 1.0008 | 639.8 | 945.3 |
| State random intercept: Delhi | 1.1597 | 0.7023 | -0.2906 | 2.5327 | 1.0007 | 653.7 | 846.6 |
| State random intercept: Haryana | -1.5895 | 0.7076 | -3.0214 | -0.1503 | 1.0010 | 649.6 | 861.9 |
| State random intercept: Jharkhand | 0.9983 | 0.6715 | -0.4039 | 2.2298 | 1.0043 | 519.4 | 693.9 |
| State random intercept: Madhya Pradesh | 0.2481 | 0.6715 | -1.1560 | 1.4558 | 1.0041 | 524.4 | 678.3 |
| State random intercept: Odisha | 0.5962 | 0.6712 | -0.8171 | 1.8129 | 1.0042 | 521.6 | 694.3 |
| State random intercept: Puducherry | 1.1223 | 0.6776 | -0.2862 | 2.3614 | 1.0043 | 509.0 | 733.3 |
| State random intercept: Punjab | -2.1658 | 0.7176 | -3.6330 | -0.7142 | 1.0015 | 644.8 | 982.9 |
| State random intercept: Rajasthan | -2.3657 | 0.6777 | -3.7561 | -1.1351 | 1.0040 | 544.1 | 858.8 |
| State random intercept: Tamil Nadu | -0.3908 | 1.2076 | -2.7709 | 2.0338 | 1.0006 | 1283.6 | 1237.0 |
| State random intercept: Uttar Pradesh | -2.0914 | 0.6988 | -3.4757 | -0.7124 | 1.0009 | 640.4 | 862.1 |
| State random intercept: Uttarakhand | -1.4268 | 0.6855 | -2.8287 | -0.1483 | 1.0038 | 531.5 | 774.0 |

**Note:** Reference categories were age 15-24 years, rural residence, no education, poorest wealth quintile, never in union, religious norms permitting alcohol use, no scheduled tribe status, and relaxed state alcohol policy. The post-lockdown coefficient applies to respondents in all reference categories. Post-lockdown interaction coefficients represent differences from this reference association. State-specific random intercepts represent deviations from the overall baseline log odds. CrI = credible interval; ESS = effective sample size; SD = standard deviation.

**Table A2.2. Posterior summaries for the Bayesian binomial logistic regression model among men**

| **Parameter** | **Posterior mean** | **Posterior SD** | **2.5th percentile** | **97.5th percentile** | **R-hat** | **Bulk ESS** | **Tail ESS** |
| --- | --- | --- | --- | --- | --- | --- | --- |
| Intercept | -0.8844 | 0.2519 | -1.3794 | -0.3906 | 1.0017 | 682.4 | 853.4 |
| Time trend | -0.2567 | 0.0203 | -0.2950 | -0.2159 | 1.0010 | 2857.3 | 1371.3 |
| Post-lockdown period | -0.1410 | 0.0756 | -0.2893 | 0.0075 | 1.0000 | 1556.3 | 1503.6 |
| Age 25-34 years (ref: 15-24) | 0.7760 | 0.0279 | 0.7196 | 0.8313 | 1.0015 | 1294.5 | 1200.2 |
| Age 35-44 years (ref: 15-24) | 0.7046 | 0.0322 | 0.6439 | 0.7683 | 1.0020 | 1323.2 | 1084.7 |
| Age 45-54 years (ref: 15-24) | 0.5286 | 0.0349 | 0.4584 | 0.5933 | 1.0012 | 1162.3 | 1176.6 |
| Urban residence (ref: rural) | 0.2127 | 0.0213 | 0.1703 | 0.2546 | 1.0025 | 2322.2 | 1544.9 |
| Primary education (ref: no education) | -0.0214 | 0.0309 | -0.0816 | 0.0390 | 1.0007 | 1842.1 | 1652.6 |
| Secondary education (ref: no education) | -0.3567 | 0.0262 | -0.4055 | -0.3058 | 0.9995 | 1405.4 | 1662.3 |
| Higher education (ref: no education) | -0.5823 | 0.0341 | -0.6489 | -0.5140 | 1.0011 | 1473.2 | 1648.2 |
| Poorer wealth quintile (ref: poorest) | -0.1910 | 0.0263 | -0.2421 | -0.1379 | 1.0016 | 1534.0 | 1256.5 |
| Middle wealth quintile (ref: poorest) | -0.3164 | 0.0284 | -0.3717 | -0.2605 | 1.0006 | 1343.4 | 1517.4 |
| Richer wealth quintile (ref: poorest) | -0.4305 | 0.0304 | -0.4897 | -0.3724 | 0.9998 | 1264.4 | 1534.7 |
| Richest wealth quintile (ref: poorest) | -0.5425 | 0.0340 | -0.6068 | -0.4779 | 1.0005 | 1292.1 | 1191.0 |
| Married/living together (ref: never in union) | 0.4766 | 0.0269 | 0.4237 | 0.5276 | 1.0001 | 1342.1 | 1480.2 |
| Divorced/separated/widowed (ref: never in union) | 0.6037 | 0.0683 | 0.4704 | 0.7356 | 1.0017 | 2048.2 | 1717.2 |
| Religious norms prohibit alcohol (ref: permit) | -1.4502 | 0.0447 | -1.5395 | -1.3673 | 1.0003 | 2708.0 | 1638.3 |
| Scheduled tribe (ref: no tribe) | 0.4055 | 0.0291 | 0.3515 | 0.4636 | 1.0002 | 2188.1 | 1408.2 |
| Temporary-prohibition state (ref: relaxed policy) | 0.4563 | 0.6014 | -0.7512 | 1.6223 | 0.9998 | 1479.2 | 1355.2 |
| Restricted-sales state (ref: relaxed policy) | 0.1275 | 0.3413 | -0.5793 | 0.8244 | 1.0024 | 698.1 | 946.0 |
| Post-lockdown x age 25-34 years | 0.1866 | 0.0575 | 0.0777 | 0.2986 | 1.0021 | 992.2 | 1121.0 |
| Post-lockdown x age 35-44 years | 0.3218 | 0.0662 | 0.1936 | 0.4568 | 1.0035 | 956.6 | 1130.3 |
| Post-lockdown x age 45-54 years | 0.3835 | 0.0690 | 0.2480 | 0.5199 | 1.0012 | 1053.9 | 956.6 |
| Post-lockdown x urban residence | -0.0231 | 0.0436 | -0.1095 | 0.0593 | 1.0007 | 2491.5 | 1722.8 |
| Post-lockdown x primary education | 0.1855 | 0.0604 | 0.0635 | 0.3037 | 1.0001 | 1640.0 | 1760.9 |
| Post-lockdown x secondary education | 0.1242 | 0.0512 | 0.0228 | 0.2267 | 0.9999 | 1431.9 | 1548.5 |
| Post-lockdown x higher education | -0.0099 | 0.0665 | -0.1365 | 0.1216 | 1.0014 | 1547.9 | 1281.5 |
| Post-lockdown x poorer wealth quintile | -0.1373 | 0.0498 | -0.2399 | -0.0436 | 0.9998 | 1478.1 | 1588.1 |
| Post-lockdown x middle wealth quintile | -0.1192 | 0.0524 | -0.2208 | -0.0145 | 1.0006 | 1608.1 | 1542.1 |
| Post-lockdown x richer wealth quintile | -0.3018 | 0.0586 | -0.4203 | -0.1949 | 1.0012 | 1256.6 | 1422.8 |
| Post-lockdown x richest wealth quintile | -0.2051 | 0.0639 | -0.3248 | -0.0775 | 1.0023 | 1282.1 | 1485.3 |
| Post-lockdown x married/living together | 0.0035 | 0.0528 | -0.0976 | 0.1093 | 0.9997 | 1236.9 | 1427.9 |
| Post-lockdown x divorced/separated/widowed | 0.0835 | 0.1279 | -0.1707 | 0.3343 | 0.9998 | 2025.7 | 1606.5 |
| Post-lockdown x religious norms prohibit alcohol | -0.2078 | 0.0998 | -0.3989 | -0.0153 | 0.9998 | 3198.9 | 1540.3 |
| Post-lockdown x scheduled tribe | 0.0905 | 0.0471 | -0.0017 | 0.1834 | 1.0002 | 2290.8 | 1495.6 |
| Post-lockdown x temporary-prohibition state | -0.1285 | 0.0639 | -0.2544 | -0.0021 | 1.0002 | 3371.7 | 1679.5 |
| Post-lockdown x restricted-sales state | 0.0868 | 0.0363 | 0.0173 | 0.1562 | 0.9997 | 2892.4 | 1697.5 |
| State-level random-intercept SD | 0.6196 | 0.1601 | 0.4038 | 1.0097 | 1.0008 | 631.2 | 896.2 |
| State random intercept: Arunachal Pradesh | 0.6902 | 0.2476 | 0.2089 | 1.1951 | 1.0002 | 678.4 | 772.5 |
| State random intercept: Chhattisgarh | 0.2896 | 0.2481 | -0.2118 | 0.7972 | 1.0002 | 671.7 | 798.2 |
| State random intercept: Delhi | 0.0666 | 0.2499 | -0.4274 | 0.5761 | 1.0005 | 676.0 | 738.2 |
| State random intercept: Haryana | -0.4098 | 0.2491 | -0.8973 | 0.0874 | 0.9999 | 685.3 | 809.1 |
| State random intercept: Jharkhand | 0.3352 | 0.2501 | -0.1629 | 0.8424 | 1.0021 | 673.8 | 854.0 |
| State random intercept: Madhya Pradesh | -0.4376 | 0.2499 | -0.9479 | 0.0514 | 1.0023 | 681.3 | 872.9 |
| State random intercept: Odisha | 0.1606 | 0.2513 | -0.3424 | 0.6609 | 1.0010 | 670.9 | 910.1 |
| State random intercept: Puducherry | 0.4291 | 0.2545 | -0.0751 | 0.9144 | 1.0023 | 672.9 | 851.7 |
| State random intercept: Punjab | 0.1008 | 0.2485 | -0.3959 | 0.5980 | 0.9999 | 676.4 | 835.3 |
| State random intercept: Rajasthan | -1.1106 | 0.2507 | -1.6057 | -0.6207 | 1.0024 | 682.7 | 875.8 |
| State random intercept: Tamil Nadu | 0.0863 | 0.5765 | -1.0274 | 1.2854 | 1.0000 | 1738.7 | 1130.9 |
| State random intercept: Uttar Pradesh | -0.6805 | 0.2478 | -1.1805 | -0.1720 | 1.0001 | 665.9 | 809.6 |
| State random intercept: Uttarakhand | 0.4613 | 0.2516 | -0.0429 | 0.9743 | 1.0019 | 686.1 | 950.1 |

**Note:** Reference categories were age 15-24 years, rural residence, no education, poorest wealth quintile, never in union, religious norms permitting alcohol use, no scheduled tribe status, and relaxed state alcohol policy. The post-lockdown coefficient applies to respondents in all reference categories. Post-lockdown interaction coefficients represent differences from this reference association. State-specific random intercepts represent deviations from the overall baseline log odds. CrI = credible interval; ESS = effective sample size; SD = standard deviation.

The post-lockdown coefficient in Tables A2.1 and A2.2 should not be interpreted as the overall adjusted odds ratio reported in the main manuscript because it applies only to the reference subgroup. Overall and subgroup-specific odds ratios were obtained from standardized predicted probabilities that incorporated the post-lockdown coefficient, relevant interaction terms, and the observed distribution of respondent characteristics.

**Table A2.3. Model diagnostics**

| **Model** | **Maximum R-hat** | **Minimum bulk ESS** | **Minimum tail ESS** | **Divergent transitions** |
| --- | --- | --- | --- | --- |
| Women | 1.0096 | 509.0 | 666.3 | 0 |
| Men | 1.0035 | 631.2 | 738.2 | 0 |

R-hat values were no greater than 1.01, effective sample sizes were adequate, and no divergent transitions were identified. These findings indicated satisfactory convergence and stable posterior sampling for both models.
